# Temporal Trends in Pregnancy Rate, Live Birth Rates, and Rates of Gestational Outcomes, 2012–2023

**DOI:** 10.1097/og9.0000000000000167

**Published:** 2026-04-16

**Authors:** Shalmali Bane, Fei Xu, Susanna Mitro, Heather Forquer, Jennifer M. Baker, Catherine Lee, Mara Greenberg, Michael Kuzniewicz, Monique Hedderson

**Affiliations:** Kaiser Permanente Northern California Division of Research, Pleasanton, California; Department of Epidemiology and Biostatistics, University of California San Francisco, San Francisco, California; Department of Obstetrics and Gynecology and Department of Pediatrics, Kaiser Permanente Northern California, Oakland, California; and Regional Perinatal Service Center, Kaiser Permanente Northern California, Santa Clara, California.

## Abstract

**OBJECTIVE:**

To describe trends in rates of pregnancy, live birth, and gestational outcomes and to assess variation by maternal demographic characteristics.

**METHODS:**

We conducted a retrospective cohort study of pregnancies with outcomes at a large and diverse integrated health care system from 2012 to 2023. We calculated temporal trends in unadjusted and adjusted pregnancy rates and live birth rates per 1,000 individuals 15–44 years of age, overall and stratified by age and race and ethnicity. We used quasi-Poisson regression models adjusted for age and self-reported race and ethnicity to estimate the overall percentage change. We also assessed the rates of plurality (per 1,000 pregnancies) and gestational outcomes, including stillbirth, ectopic pregnancy, molar pregnancy, miscarriage, and induced abortion (per 1,000 singleton pregnancies).

**RESULTS:**

In total, 700,159 pregnancies from 2012 to 2023 were included. The adjusted pregnancy rate declined from 78 per 1,000 individuals 15–44 years of age in 2012 to 70 in 2023 (−10.2% [95% CI, −15.6% to −4.5%]), and the adjusted live birth rate declined from 53 per 1,000 individuals 15–44 years of age in 2012 to 45 in 2023 (−13.8% [95% CI, −19.3% to −8.0%]). Among singleton pregnancies, the adjusted rates of miscarriage (2012 rate: 166.3 per 1,000 pregnancies; 2023 rate: 162.2 per 1,000 pregnancies, change −2.4% [95% CI, −4.2% to −0.6%]), molar pregnancy (2012 rate: 1.9 per 1,000 pregnancies; 2023 rate: 1.1 per 1,000 pregnancies, change −38.2% [95% CI, −52.1% to −20.2%]), and ectopic pregnancy (2012 rate: 16.7 per 1,000 pregnancies; 2023 rate: 16.3 per 1,000 pregnancies, change −10.3% [95% CI, −15.4% to −4.8%]) declined. The adjusted rate of stillbirth was relatively stable but rare (3.1 and 3.4 per 1,000 singleton pregnancies in 2012 and 2023, respectively; change 5.3% [95% CI, −7.1% to 19.3%]), whereas the adjusted rate of induced abortions increased over time by 27.3% (95% CI, 23.6% to 31.1%) from 133.2 per 1,000 pregnancies in 2012 to 172.4 per 1,000 pregnancies in 2023.

**CONCLUSION:**

In an integrated health care delivery system, the pregnancy and live birth rates declined in the past decade, mirroring national surveillance data. The greater decline in live birth rate compared with pregnancy rate may be explained by the higher rate of induced abortion because the rates of other gestational outcomes declined over time.

## Abstract 2

Pregnancy and live birth rates declined in the past decade; this decline in live births may be explained by increases in the rate of induced abortion.

Long-term trends in birth rates reflect complex societal changes, including shifts in economy factors and societal norms around parenting.^1,2^ Monitoring birth rates and pregnancy rates is important for tracking population growth and decline, economic and environmental repercussions, and the provision of health care services, especially reproductive health care services. In addition, these data are valuable for government agencies implementing policies on family planning and maternity care.

The U.S. live birth rate, defined as the number of live births per 1,000 individuals 15–44 years of age, is declining, with a historic low in 2023.^3,4^ Nationally, this decline intensified during the coronavirus disease 2019 (COVID-19) pandemic.^4,5^ Pregnancy rates, defined as the number of pregnancies per 1,000 individuals 15–44 years of age regardless of pregnancy outcome, are challenging to quantify because claims data and vital records capture only births. Given that not all pregnancies result in a live birth, capturing the relationship between pregnancy and live birth rates, particularly among sociodemographic subgroups, can provide context on reproductive decision making, quality of care, and maternal health.^6^ Only a handful of studies have documented how the rates of gestational outcomes such as stillbirth differed before and after the 2020 pandemic.^7–10^

To inform the allocation of health care system resources and patient-centered family planning support mechanisms, there is a need to understand trends in pregnancy and live birth rates, particularly how these trends have varied by maternal demographics since the COVID-19 pandemic. National surveillance statistics and trends provide context; however, limited sources have information on pregnancy rates or outcomes such as induced abortion. To address this gap, we used data from Kaiser Permanente Northern California, a large and diverse integrated health care delivery system, to describe overall trends in pregnancy and live birth rates from 2012 to 2023. We also sought to describe trends in these rates by maternal demographic characteristics and select gestational outcomes (plurality defined as the number of fetuses for a given pregnancy, stillbirth, ectopic pregnancy, molar pregnancy, miscarriage, and induced abortion).

## METHODS

Kaiser Permanente Northern California provides health care to more than 4.6 million individuals representing 30% of the population living in the service area (which spans 14 counties of the greater Bay Area and the California Central Valley from Sacramento to Fresno). The population is representative of the demographic characteristics of the entire population from this geographic area.^11–13^ Kaiser Permanente Northern California is vertically integrated such that all care is provided in a closed system and documented in the electronic health record (EHR). As clinical records, the EHRs are robust in data quality and completeness.^14^

The Kaiser Permanente Northern California Perinatal Research Unit Obstetric Database is a comprehensive database including pregnancy episodes with onset beginning in 2011 and associated live-born infant data; the database is updated monthly. The database includes EHR and administrative data. For a pregnancy episode to be included in the database, the pregnancy episode must have a known pregnancy onset date, gestational age, gestational outcome, and singleton or multiple gestation status. Further data quality checks are conducted according to specific criteria (eg, manual verification is conducted for episodes with clinically implausible outcomes based on gestational age). Records are indexed on unique pregnancy episodes identified with the Center for Effectiveness and Safety Research Pregnancy Outcome Episode Table and Kaiser Permanente Northern California's Neonatal Minimum Dataset and Infant Cohort.^15,16^

Each pregnancy episode includes data obtained from the EHR on pregnancy onset date, gestational outcome date, gestational outcomes (Appendix 1, available online at http://links.lww.com/AOG/E625; gestational outcomes are defined as the outcome of the pregnancy, ie, whether it resolved in a live birth, ectopic pregnancy, molar pregnancy, stillbirth, miscarriage, or induced abortion), maternal characteristics (eg, age, body mass index [BMI], parity), any maternal diagnoses (eg, gestational diabetes or gestational hypertension), locations of live births, maternal delivery hospitalization data (admission and discharge date/times, delivery mode, delivery medications, delivery complications), and antenatal and postpartum care (clinic encounters, postpartum readmissions, postpartum emergency department visits). The infant data include infant demographics, infant hospitalization data, and infant characteristics such as gestational age and birth weight. Pregnancies resulting in a live birth are linked to the infant medical record number to facilitate linkage to neonatal data. Mode of delivery has been validated against the California birth certificate record and the Kaiser Permanente Northern California infant cohort database (approximately 98.9% were validated from at least two sources).^15^ In addition, the Neighborhood Deprivation Index value, a composite indicator of area-level socioeconomic status based on census tract, is available for most records.^17^ To define gestational outcomes, including induced abortion, miscarriage, molar pregnancies, and ectopic pregnancies, a comprehensive and multistep algorithm is used that integrates EHR obstetric data, the Epic Stork module, virtual data warehouse diagnosis, procedure, medication, and local data sources. Some of the methods, including International Classification of Diseases 9th and 10th Revision codes, Diagnosis-Related Group codes, and medication names, are listed in Appendix 2 (http://links.lww.com/AOG/E625). Gestational outcomes are determined through a hierarchical process that incorporates all available information, with validation and refinement steps applied to enhance accuracy. The algorithm also systematically excludes weak or conflicting information to ensure that each pregnancy episode reflects a clinically coherent and validated outcome.

This retrospective cohort study included eligible pregnancies from the Kaiser Permanente Northern California EHR with an outcome date between 2012 and 2023, identified with the described previously database (Appendix 3, http://links.lww.com/AOG/E625). Pregnancies were excluded if they did not have Kaiser Permanente Northern California membership or use at the time of both pregnancy onset and gestational outcome. All eligible pregnancies, including multiple births to the same individuals, were included in the analysis of trends in pregnancy and live birth rates. For the analysis of trends, pregnancies with missing adjustment factors (0.1% of the overall sample, with 800 observations missing Neighborhood Deprivation Index score and 4 observations missing maternal age) were excluded because of insufficient observations for adjustment; in addition, those in age groups of less than 15 years or more than 44 years (n=4,415) were excluded given the small numbers in these categories and to be comparable with prior national studies. All pregnancies with multiple gestations were excluded from the analysis of gestational outcomes trends to avoid confounding resulting from high risks of adverse gestational outcomes in multiple gestations.

First, we described temporal trends in unadjusted pregnancy rate and live birth rate per 1000 Kaiser Permanente Northern California members who were individuals 15–44 years of age. The denominator included all individuals classified as female based on sex assigned at birth in the Kaiser Permanente Northern California EHR; however, to be inclusive, we refer to this population as individuals 15–44 years of age henceforth. These trends are also presented stratified by demographic characteristics: age (15–19, 20–24, 25–29, 30–34, 35–39, 40–44 years) and self-reported race and ethnicity (Asian, Black, Hispanic, White, additional races and ethnicities; additional races and ethnicities include Native American, Pacific Islander, and multiracial individuals).

Next, we estimated the directly adjusted rate for each year. This rate was adjusted to reflect the average demographic distribution of the population across the study period, ie, adjusted for age (categorical) and race and ethnicity. Adjusted quasi-Poisson regression models (that can account for both underdispersion and overdispersion) were used to estimate the percentage of change over the entire study period (2012–2023) and 95% CI, including adjustments for common, strong confounders that are associated with outcomes of interest: age (categorical), race and ethnicity and year. Self-reported race and ethnicity were considered a socially constructed factor created by racist historical processes (eg, slavery) in the United States that persist in our contemporary context.^18^

Lastly, to describe temporal trends in rates of gestational outcomes, we present the directly adjusted rate of a given outcome 1,000 singleton pregnancies (live birth, ectopic pregnancy, molar pregnancy, stillbirth, miscarriage, induced abortion). We additionally present the directly adjusted rate for plurality as per 1,000 pregnancies (plurality: singleton, twins or triplets) among all pregnancies, including multiples. In addition, we provide the percentage change over the study period and 95% CI from quasi-Poisson regression models that were adjusted for age (categorical, 15–19, 20–24, 25–29, 30–34, 35–39, 40–44 years), BMI (categorical, below 25, 25.0–29.9, 30 and above, missing), Neighborhood Deprivation Index score quartile (defined from the overall Kaiser Permanente Northern California population per year), self-reported race and ethnicity (Asian, Black, Hispanic, White, additional races and ethnicities, and missing), and year; the goal of adjustment was to account for the shift in the population demographics over the study period.

The Kaiser Permanente Northern California IRB for the Protection of Human Subjects approved this study. The requirement for informed consent was waived. The analysis was conducted with R z4.1.2.

## RESULTS

Among 700,159 pregnancies from 2012 to 2023, the average age was 31 years (Table 1). Most pregnancies were in individuals with commercial insurance (85.8%), and 38.7% were in nulliparous individuals. Of the total pregnancies, 66.7% resulted in live births, and 17% were classified as miscarriages, 14% as induced abortions, and less than 2% as ectopic pregnancies, molar pregnancies, or stillbirths. Table 1 also shows that the distribution of covariates shifted over the study period from the earlier (2012–2019) to later (2020–2023) years.

**Table 1.** Characteristics of Pregnancy Episodes From 2012 to 2023 at Kaiser Permanente Northern California

Characteristics and Gestational Outcomes	2012–2023 Cohort (N=700,159)	2012–2019 Cohort (N=447,619)	2020–2023 Cohort (N=252,540)
Age, mean±SD (y)	31±5.9	31±5.9	32±5.7
Age, categorical*			
10–14 y	195 (less than 0.1)	143 (less than 0.1)	52 (less than 0.1)
15–19 y	19,656 (2.8)	14,551 (3.3)	5,105 (2.0)
20–24 y	83,998 (12.0)	57,426 (12.8)	26,572 (10.5)
25–29 y	161,030 (23.0)	105,388 (23.5)	55,642 (22.0)
30–34 y	233,242 (33.3)	147,002 (32.8)	86,240 (34.1)
35–39 y	153,747 (22.0)	93,534 (20.9)	60,213 (23.8)
40–44 y	44,063 (6.3)	26,919 (6.0)	17,144 (6.8)
45+ y	4,224 (0.6)	2,653 (0.6)	1,571 (0.6)
Prepregnancy BMI, mean±SD	27.1±6.4	26.8±6.3	27.8±6.6
BMI category			
Below 25	295,132 (42.2)	200,551 (44.8)	94,581 (37.5)
25.0–29.9	187,527 (26.8)	118,525 (26.5)	69,002 (27.3)
30 and above	178,261 (25.5)	105,407 (23.5)	72,854 (28.8)
Missing	39,239 (5.6)	23,136 (5.2)	16,103 (6.4)
Race and ethnicity			
Asian	153,924 (22.0)	95,488 (21.3)	58,436 (23.1)
Black	59,067 (8.4)	37,556 (8.4)	21,511 (8.5)
Hispanic	188,305 (26.9)	114,983 (25.7)	73,322 (29.0)
White	237,740 (34.0)	159,214 (35.6)	78,526 (31.1)
Additional races and ethnicities	46,940 (6.7)	33,636 (7.5)	13,304 (5.3)
Missing	14,183 (2.0)	6,742 (1.5)	7,441 (2.9)
Parity			
0	271,042 (38.7)	175,291 (39.2)	95,751 (37.9)
1	224,752 (32.1)	143,735 (32.1)	81,017 (32.1)
2+	157,408 (22.5)	100,265 (22.4)	57,143 (22.6)
Missing	46,957 (6.7)	28,328 (6.3)	18,629 (7.4)
Insurance			
Medicare/Medicaid	79,594 (11.4)	45,927 (10.3)	33,667 (13.3)
Commercial	600,958 (85.8)	387,533 (86.6)	213,425 (84.5)
Other/missing	19,607 (2.8)	14,159 (3.2)	5,448 (2.2)
NDI score			
Least deprived	145,939 (20.8)	96,108 (21.5)	49,831 (19.7)
Second quartile	187,772 (26.8)	120,205 (26.9)	67,567 (26.8)
Third quartile	196,950 (28.1)	124,779 (27.9)	72,171 (28.6)
Most deprived	168,698 (24.1)	105,937 (23.7)	62,761 (24.9)
Missing	800 (0.1)	590 (0.1)	210 (0.1)
Plurality			
Singleton	691,654 (98.8)	441,733 (98.7)	249,921 (99.0)
Twin	8,346 (1.2)	5,760 (1.3)	2,586 (1.0)
Triplet+	159 (less than 0.1)	126 (less than 0.1)	33 (less than 0.1)
Outcome			
Ectopic	12,415 (1.8)	8,134 (1.8)	4,281 (1.7)
Live born	466,742 (66.7)	301,139 (67.3)	165,603 (65.6)
Molar	839 (0.1)	563 (0.1)	276 (0.1)
Miscarriage	119,263 (17.0)	75,837 (16.9)	43,426 (17.2)
Stillborn	2,528 (0.4)	1,568 (0.4)	960 (0.4)
Induced abortion	98,318 (14.0)	60,357 (13.5)	37,961 (15.0)
Pregnancies with multiples with different gestational outcomes	54 (less than 0.1)	21 (less than 0.1)	33 (less than 0.1)

BMI, body mass index; NDI, Neighborhood Deprivation Index.

Values are n (%) unless indicated otherwise.

* Four individuals were missing data on age.

**Fig. 1.**
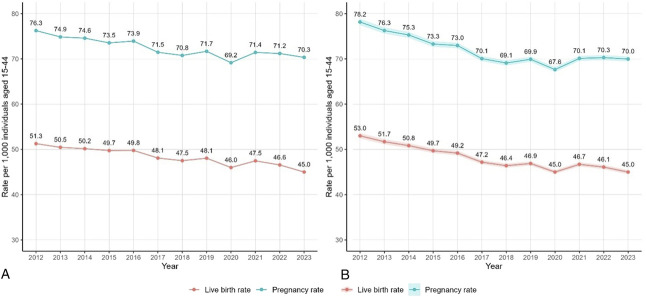
Time trends in pregnancy rate and live birth rate per 1,000 individuals, unadjusted **(A)** and adjusted **(B)**: Kaiser Permanent Northern California, 2012–2023. Adjusted for race and ethnicity and categorical age.

Pregnancy and live birth rates declined over time (Fig. 1). The unadjusted pregnancy rate was 76 per 1,000 individuals 15–44 years of age in 2012 and declined to 70 in 2023. Similarly, live birth rates declined from 51 per 1,000 individuals 15–44 years of age in 2012 to 45 in 2023. The percentage change in the adjusted pregnancy rate and live birth rate over the study period was −10.2% (95% CI, −15.6 to −4.5) and −13.8% (95% CI, −19.3 to −8.0), respectively. Adjusted pregnancy rates dropped to their lowest in 2020 (67.6 per 1,000 individuals 15–44 years of age) but in subsequent years rates returned to similar levels as in prior years (eg, 70.0 per 1,000 individuals 15–44 years of age in 2023). Adjusted live birth rates dropped to their lowest in 2020 (45.0 per 1,000 individuals 15–44 years of age), increasing slightly in 2021 (46.7 per 1,000 individuals 15–44 years of age), and decreasing again to their lowest levels in 2023 (45.0 per 1,000 individuals 15–44 years of age).The overall trends of declining pregnancy and live birth rates remained after adjustment.

*Bane. Trends in Pregnancy and Gestational Outcomes. O&G Open 2026*.

**Fig. 2.**
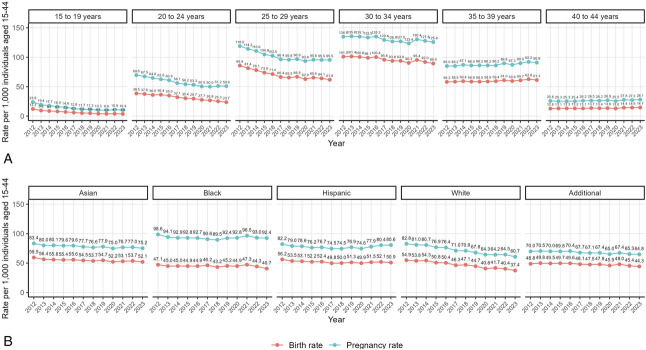
Trends in pregnancy rate and birth unadjusted rate per 1,000 individuals, stratified by sociodemographic characteristics: Kaiser Permanente Northern California, 2012–2023. Pregnancy and birth rates by maternal age **(A)** and pregnancy and birth rates by race and ethnicity **(B)**.

Stratified by age (Fig. 2A), trends in unadjusted pregnancy and live birth rates decreased over time for younger age groups (eg, 24 in 2012 vs 10 in 2023 for pregnancy rate among individuals 15–19 years of age) and increased slightly for older age groups (eg, 26 in 2012 vs 28 in 2023 for pregnancy rate among individuals 40–44 years of age). The 25- to 29-year and 30- to 34-year age groups were the only subgroups in whom pregnancy and live birth rates in 2021 exceeded several years prior (eg, 127 in 2018 compared with 130 in 2021 per 1,000 individuals); the overall decline in the 30- to 34-year age group was smaller compared with the younger groups. Pregnancy and live birth rates trended downward over time for all racial and ethnic groups (Fig. 2B); the only exception was that pregnancy rates consistently increased since 2020 among Hispanic individuals (78 in 2021 vs 81 in 2023). Notably, Black individuals had higher pregnancy rates and lower live birth rates relative to other racial and ethnic subgroups. In addition, the steepest decline in pregnancy and live birth rates were reported among White individuals (eg, from 83 in 2012 to 61 in 2023 for pregnancy rate).

*Bane. Trends in Pregnancy and Gestational Outcomes. O&G Open 2026*.

**Fig. 3.**
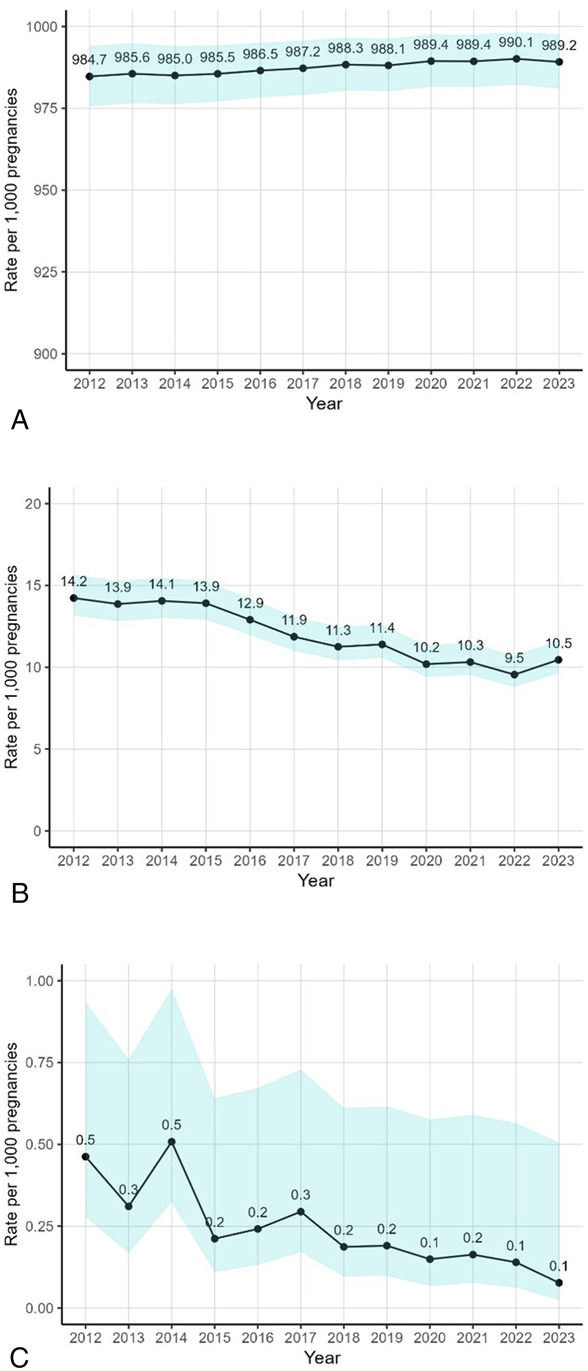
Trends in adjusted rates of plurality per 1,000 pregnancies: Kaiser Permanente Northern California, 2012–2023.* Singleton **(A)**, twin **(B)**, and triplet or higher order **(C)**. *Adjusted for categorical age, race and ethnicity, categorical body mass index, Neighborhood Deprivation Index score quartile, and year. *Shaded regions* indicate 95% confidence bounds.

**Fig. 4.**
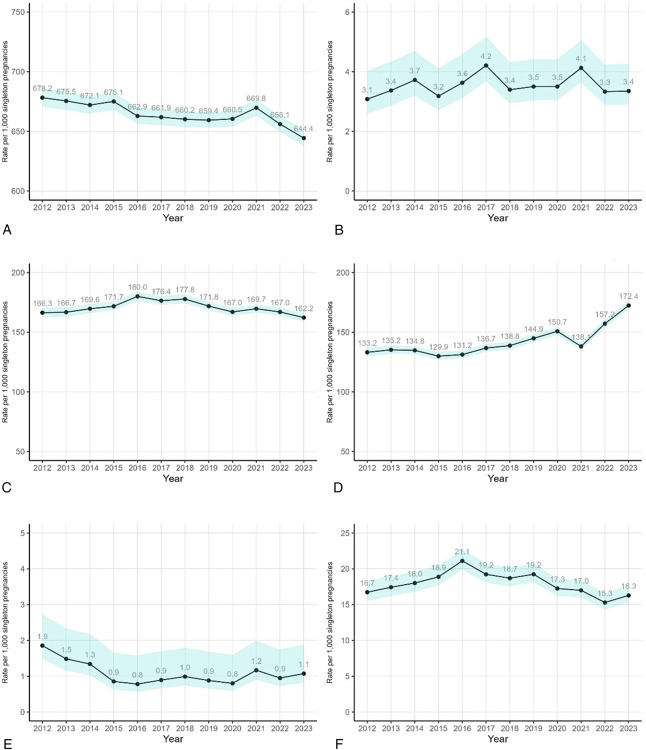
Trends in adjusted rates of gestational outcomes per 1,000 singleton pregnancies: Kaiser Permanente Northern California, 2012–2023.* Live birth **(A)**, stillbirth **(B)**, miscarriage **(C)**, induced abortion **(D)**, molar pregnancy **(E)**, and ectopic pregnancy **(F)**. *Adjusted for categorical age, race and ethnicity, categorical body mass index, Neighborhood Deprivation Index score quartile, and year. *Shaded regions* indicate 95% confidence bounds.

The rate of singletons per 1,000 pregnancies increased slightly (2012 rate: 984.7 per 1,000 pregnancies; 2023 rate: 989.2 per 1,000 pregnancies, adjusted change 0.5% [95% CI, 0.5% to 0.6%]), whereas the rate of multiples declined over time for twins (2012 rate: 14.2; 2023 rate: 10.5, change −35.1% [95% CI, −39.5% to −30.3%]) and for triplets (2012 rate: 0.5; 2023 rate: 0.1, change −76.3% [95% CI, −86.6% to −58.0%]; Fig. 3). Among singleton pregnancies, the rate of live births decreased slightly over time (2012 rate: 678.2 per 1,000 pregnancies; 2023 rate: 644.4 per 1,000 pregnancies, change −4.3% [95% CI, −5.3% to −3.2%]; Fig. 4), whereas the rate of stillbirth was relatively stable (5.3% [95% CI, −7.1% to 19.3%]) and remained relatively rare (3.1 and 3.4 per 1,000 singleton pregnancies in 2012 and 2023, respectively). The rates of miscarriage and ectopic pregnancies declined slightly over time (2012 rate: 166.3 per 1,000 pregnancies; 2023 rate: 162.2 per 1,000 pregnancies, change −2.4% [95% CI, −4.2% to −0.6%]; and 2012 rate: 16.7 per 1,000 pregnancies; 2023 rate: 16.3 per 1,000 pregnancies, change −10.3% [95% CI, −15.4% to −4.8%]), whereas molar pregnancies decreased substantially (2012 rate: 1.9 per 1,000 pregnancies; 2023 rate: 1.1 per 1,000 pregnancies, change −38.2% [95% CI, −52.1% to −20.2%]). The rate of induced abortions increased over time by 27.3% (95% CI, 23.6–31.1%) from 133.2 per 1,000 pregnancies in 2012 to 172.4 per 1,000 pregnancies in 2023.

*Bane. Trends in Pregnancy and Gestational Outcomes. O&G Open 2026*.

*Bane. Trends in Pregnancy and Gestational Outcomes. O&G Open 2026*.

## DISCUSSION

We examined a cohort of 700,159 pregnancies in a large integrated health care delivery system in Northern California from 2012 to 2023. The rates of pregnancy and live birth declined over time; the decline was steepest in 2020, likely because of the COVID-19 pandemic. These rates declined across all age groups among individuals less than 35 years of age, whereas rates increased among individuals 35 years of age and older. In general, pregnancy and live birth rates declined for all racial and ethnic subgroups (especially among White individuals) with the exception of increasing pregnancy rates after 2020 among Hispanic individuals. Among singleton pregnancies, the rates of miscarriage, ectopic pregnancy, and molar pregnancy declined. Although the rate of stillbirth was relatively constant, the rate of induced abortions increased, particularly after 2021.

The downward trend in live birth rate in the Kaiser Permanente Northern California population mirrors declining trends at the national level.^3^ However, the live birth rates nationally per 1,000 individuals 15–44 years of age (56 and 54 for 2022 and 2023, respectively) are higher than the adjusted rates among this study population of insured individuals from California (47 and 45, respectively). Our data also align with a nationally observed dip in the live birth rate in 2020, potentially attributable to the COVID-19 pandemic.^3^ Several pandemic-related factors may have contributed, including declining mental and physical health, food insecurity, unemployment, challenges with childcare, and policies limiting in-person interactions; these eased in the subsequent years.^19^ Limited existing data beyond 2021 suggest that live birth rates in 2022 were similar to rates in 2021 and that live birth rates declined by 3% between 2022 and 2023.^20,21^ Calculating population-level pregnancy rates is challenging; existing studies combine births, abortions, and fetal loss counts from multiple sources.^22–24^ Given our setting in a closed, integrated system, we can accurately capture pregnancies. These results show that trends in pregnancy rate and live birth rate closely parallel each other, similar to national data on pregnancy rates that are available until 2019.^24^

The steady decline in pregnancy and live birth rates was largely among teens and younger adults (younger than 30 years of age), whereas the pregnancy and live birth rates increased over time for adults 35 years of age and older, consistent with national trends.^3,20,21^ Individuals 30–34 years of age were the only subgroup among whom the pregnancy and live birth rates declined in 2020, with a rebound in 2021 that exceeded rates in 2019. We hypothesize that this represents planned pregnancies that were delayed during the height of the pandemic.

Hispanic individuals were the only group whose pregnancy rates did not resume a downward trend, consistent with California vital statistics showing that the 2021 rise in births was driven by younger, highly educated Hispanic individuals.^19^ White populations experienced the largest declines in pregnancy and live birth rates over time, mirroring national patterns.^24^ Black individuals had higher rates of pregnancy but lower live birth rates compared with other subgroups throughout the study period. Nationally, Black individuals experience higher rates of unintended pregnancy and adverse perinatal outcomes, disparities that are attributed largely to structural racism and barriers to care.^25–32^ Notably, these patterns persisted in our insured population with access to health care services. Although all members at Kaiser Permanente Northern California have comparable access to contraceptive care, future research should determine whether racial and ethnic subgroups within integrated health care systems receive equitable, high-quality, and culturally response reproductive care.^33,34^

In addition to the decline in the live birth rate among reproductive-aged individuals, the rates of other gestational outcomes among 1,000 singleton pregnancies either stayed the same or declined over time. A national study found that the rate of live births decreased by 5.2% during the pandemic (2020–2021).^7^ In contrast, we report that the rate of live births increased between 2020 and 2021 (from 660 to 670 per 1,000 singleton pregnancies), although the rate declined in the subsequent years. This is attributable partly to the increased induced abortion rates over time, with a brief decline between 2020 and 2021 and steeper increase in 2023. Corresponding data show increases in induced abortion rates starting from 2018 at a national level.^35–37^ Furthermore, survey data from California report a substantial surge in abortion volume from 2022 to 2023.^38^ This could be an indirect reflection of the restrictive abortion policies in states outside of California from the 2022 *Dobbs v Jackson* ruling. Potential reasons for this include individuals with California coverage who have temporarily relocated, returning to the state in response to reproductive health policies, and care from independent abortion clinics referred to Kaiser Permanente Northern California clinics that are at capacity because of increased out-of-state individuals. Rates of molar pregnancy, a rare outcome, declined substantially over time; reasons for this are unclear, but these findings are in line with older studies documenting an overall decline in gestational trophoblastic diseases.^39^ We also report a decline in multiple births per 1,000 pregnancies, which is also observed nationally. This likely reflects practice changes for assisted reproduction technology; single-embryo transfers are increasingly recommended (6–64% increase among assisted reproduction technology–conceived infants from 2000 to 2017), resulting in a decrease in multiple births.^40–43^

There is a need to understand how drivers of trends over time in pregnancy and live birth rates vary among demographic subgroups, particularly through qualitative research, given the highly personal, cultural, and social factors behind individual decision making in fertility and conception. In particular, the widened disparity between pregnancy and live birth rates among Black individuals represents an important area of future inquiry, particularly to understand the lived experiences of Black birthing people, including experiences of structural racism.

The strengths of this study include the use of a large, diverse population from a large, integrated delivery system that has previously not been analyzed for pregnancy and birth trends in the past decade. This comprehensive database allowed us to examine all potential outcomes of pregnancy, including rare gestational outcomes such as ectopic and molar pregnancies, that are not typically widely investigated with sufficient sample size and detail. In addition, we are able comment on trends in rarer outcomes (eg, miscarriage and induced abortion) in relation to the live birth rate within a single health system.

Our findings should be interpreted in the context of our analytic decisions. For example, we examined only pregnancies to individuals 15–44 years of age in the numerator of our rates. In addition, as the stratified results demonstrate, overall trends can mask trends among stratified subgroups, so findings should be interpreted in that context. By nature of using data from an integrated health care system, we consider only insured patients with a mix of public and private insurance. In general, our insured population is representative of the underlying population in the area given that Kaiser Permanente provides care for 30% of the underlying population.^11,12^ However, we are unable to assess how including an uninsured population would affect these trends. Thus, our population of health system members may not be representative of all populations. Second, we report pregnancy rates per 1,000 individuals 15–44 years of age; however, it is unlikely that we captured all pregnancies. For example, cases of miscarriage before contact with the health care system or induced abortions performed outside of the health system may be underrepresented in this dataset. It is possible that this could result in ascertainment bias. However, given the closed and capitated nature of the Kaiser Permanente Northern California health system, we expect most patients to opt to seek abortion care within the health system. Third, we opted to exclude 804 individuals with missing data for Neighborhood Deprivation Index score and age because there were insufficient observations to form their own informative category. It is possible that the exclusion of these individuals led to potential selection bias; however, given that this is a relatively small proportion of the overall sample (0.1%), it is unlikely that inclusion would alter our findings. As an obstetric database, the Perinatal Research Unit Obstetric Database is also subject to limitations common to EHR systems that can limit the research applicability of such databases.

The trends in pregnancy and live birth rates in an integrated delivery health care system mirror national surveillance data. There are variations in pregnancy and live birth rates by maternal demographic characteristics such as age and race and ethnicity. There was an overall decline in the rate of live births and gestational outcomes such as stillbirth, offset by increasing rates of induced abortion. Trends in pregnancy and birth rates should be considered when developing health care policies and matching resource allocation to identified needs for the general population.
